# Perchloride of Mercury

**Published:** 1894-03-03

**Authors:** 


### NEW DRUGS AND PREPARATIONS.

[We shall be glad to receive, at our Office, 428, Strand, London, W.C., from the manufacturers, specimens of all new preparations and drugs which may be brought out from time to time.]

### PERCHLORIDE OF MERCURY.

Maurel[1] has made an exhaustive research on the action of this drug on blood corpuscles. In large amounts it affects both varieties, in small quantities only the white are influenced. The minimal fatal quantity for any organism is the quantity necessary to kill the white corpuscles, and no histological element is so susceptible as these. In the usual doses in which it is given by the mouth it acts upon the white corpuscles only, but when injected subcutaneously or into the muscles it acts on both. Its antiseptic action varies very much according to the particular variety of micro-organism; thus it is efficacious against staphyloccus albus in a strength of 1 to 5,000, while in the case of the bacillus of carbuncle 1 in 80,000 is sufficient. Considering its poisonous action on white blood corpuscles it should only be used in such diseases as those in which it acts on the micro-organism more easily than on white corpuscles. These in man begin to be acted upon by solutions of 1 in 60,000, but this proportion varies in different animals. Jemma,[2] thinking that in many bacterial diseases the cause lies in the blood, has injected perchloride of mercury directly into the veins. No definite conclusions can be drawn as his cases are too few, but in all 300 injections were given without any bad result. The dose was usually five milligrammes. He believes that this is the best way to administer mercury, and that it is more painless than subcutaneous or intramuscular injections. Froloff[3] finds salicylate of mercury as efficacious as the perchloride.

[1] Therapeutic Gazette, Aug. 15, 1893. [2] Gazz. d. Osp., June 10, 1893. [3] Vratch, No. 13, 1893.

